# Neuromelanin Imaging and Dopaminergic Loss in Parkinson's Disease

**DOI:** 10.3389/fnagi.2016.00196

**Published:** 2016-08-22

**Authors:** Ioannis U. Isaias, Paula Trujillo, Paul Summers, Giorgio Marotta, Luca Mainardi, Gianni Pezzoli, Luigi Zecca, Antonella Costa

**Affiliations:** ^1^Department of Neurology, University Hospital WuerzburgWürzburg, Germany; ^2^Centro Parkinson, Pini-CTOMilan, Italy; ^3^Department of Neuroradiology, Fondazione IRCCS Ca' Granda Ospedale Maggiore PoliclinicoMilan, Italy; ^4^Department of Electronics, Information and Bioengineering, Politecnico di MilanoMilan, Italy; ^5^Department of Nuclear Medicine, Fondazione IRCCS Ca' Granda Ospedale Maggiore PoliclinicoMilan, Italy; ^6^Italian National Research Council, Institute of Biomedical TechnologiesSegrate, Italy

**Keywords:** neuromelanin, dopamine, Parkinson's disease, MRI, FP-CIT SPECT

## Abstract

Parkinson's disease (PD) is a progressive neurodegenerative disorder in which the major pathologic substrate is a loss of dopaminergic neurons from the substantia nigra. Our main objective was to determine the correspondence between changes in the substantia nigra, evident in neuromelanin and iron sensitive magnetic resonance imaging (MRI), and dopaminergic striatal innervation loss in patients with PD. Eighteen patients and 18 healthy control subjects were included in the study. Using neuromelanin-MRI, we measured the volume of the substantia nigra and the contrast-to-noise-ratio between substantia nigra and a background region. The apparent transverse relaxation rate and magnetic susceptibility of the substantia nigra were calculated from dual-echo MRI. Striatal dopaminergic innervation was measured as density of dopamine transporter (DAT) by means of single-photon emission computed tomography and [^123^I] N-ω-fluoropropyl-2b-carbomethoxy-3b-(4-iodophenyl) tropane. Patients showed a reduced volume of the substantia nigra and contrast-to-noise-ratio and both positively correlated with the corresponding striatal DAT density. The apparent transverse relaxation rate and magnetic susceptibility values of the substantia nigra did not differ between patients and healthy controls. The best predictor of DAT reduction was the volume of the substantia nigra. Clinical and imaging correlations were also investigated for the locus coeruleus. Our results suggest that neuromelanin-MRI can be used for quantifying substantia nigra pathology in PD where it closely correlates with dopaminergic striatal innervation loss. Longitudinal studies should further explore the role of Neuromelanin-MRI as an imaging biomarker of PD, especially for subjects at risk of developing the disease.

## Introduction

Parkinson's disease (PD) is characterized by a loss of neuromelanin (NM) containing dopaminergic neurons in the substantia nigra (SN), with a consequent reduction of dopamine concentration in the putamen and caudate nucleus (Ehringer and Hornykiewicz, 1998), as well as iron deposition throughout the SN. NM-containing neurons of the locus coeruleus (LC) also degenerate early in the disease process (Braak et al., 2003; Zarow et al., 2003). It has been suggested that for every PD patient who presents with motor signs there may be 10 subclinical cases in the community (Golbe, 1993), as destruction of 60% of the NM-laden neurons in the SN is expected before motor symptoms are manifest (Fearnley and Lees, 1991). Identifying individuals in the preclinical stage of PD is a fundamental step if we aim to reveal the pathophysiological mechanisms of PD and consequently develop strategies to delay disease onset.

To date, imaging measures of dopaminergic function, such as Single-photon emission computed tomography (SPECT) with [I-123] N-ω-fluoropropyl-2b-carbomethoxy-3b-(4-iodophenyl) tropane (FP-CIT), represent a robust biomarkers for early PD detection and assessing disease progression (Isaias et al., 2006, 2007; de la Fuente-Fernández et al., 2011). Such markers, however, are mainly indicative of dopamine nerve terminal activity which could be altered by compensatory mechanisms, both endogenous and in response to treatment (Brooks and Pavese, 2011; Stoessl et al., 2014) rather than SN cell counts.

MRI techniques sensitive to NM (Sasaki et al., 2006; Kitao et al., 2013; Miyoshi et al., 2013; Ogisu et al., 2013; Ohtsuka et al., 2014) and iron (Graham et al., 2000; Martin et al., 2008; Baudrexel et al., 2010; Schweser et al., 2011; Lotfipour et al., 2012; Ulla et al., 2013) have been found to provide information about SN degeneration with potential applications as biomarkers of PD (Kashihara et al., 2011; Schwarz et al., 2011; Matsuura et al., 2013; Miyoshi et al., 2013; Castellanos et al., 2015). NM containing structures can be identified in the NM-sensitive images (NM-MRI) as areas of hyperintensity. A direct comparison between post-mortem NM-MRI and neuropathological findings has shown that NM-MRI signal intensity in the SN is closely associated with the quantity of NM-containing neurons (Kitao et al., 2013), supporting the link between NM-MRI hyperintensity with the presence of NM.

Several histopathological, biochemical and *in-vivo* brain imaging studies have shown an increase in total iron concentration in the SN of PD patients (Dexter et al., 1987; Graham et al., 2000; Zecca et al., 2001, 2004; Martin et al., 2008; Baudrexel et al., 2010). Iron in tissue has an effect on the apparent transverse relaxation rates (R2^*^) that has been widely exploited to characterize iron deposition in the SN (Baudrexel et al., 2010; Ulla et al., 2013; Barbosa et al., 2015). The paramagnetic properties of iron also influence the magnetic susceptibility of the tissues (Schweser et al., 2011). This has recently been demonstrated to allow quantification of tissue iron content based on measurements of magnetic field inhomogeneity through a technique called quantitative susceptibility mapping (QSM) (Schweser et al., 2011; Langkammer et al., 2012; Lotfipour et al., 2012) or visualized qualitatively as susceptibility weighted images (Rossi et al., 2010; Schwarz et al., 2014; Langley et al., 2015; Reiter et al., 2015).

This study aimed to investigate the correlations between MRI markers (NM-MRI, R2^*^, susceptibility) of the SN and LC of PD patients and the corresponding nigro-striatal dopaminergic innervation loss as measured by SPECT with FP-CIT.

## Subjects and methods

### Subjects

The study involved 18 subjects with PD (13 males; median age: 64 years, range: 46–77 years) and 18 age-matched healthy controls (HC group; 11 males; median age: 58 years, range: 47–77 years). The diagnosis of PD was made according to the UK Parkinson Disease Brain Bank criteria. The disease stage was determined using the Hoehn and Yahr scale and the disease severity was evaluated using the Unified Parkinson Disease Rating Scale (UPDRS). UPDRS akinetic-rigid score (UPDRSAK) was derived from the sum of UPDRS items 22 [head item excluded]-23-24-25-26. We calculated this UPDRS sub-score as putaminal FP-CIT binding was shown to exclusively correlate with the extent of rigidity and hypokinesia (Isaias et al., 2007). None of the patients showed signs indicative of atypical Parkinsonism over a period of at least 3 years prior to the enrolment to this study. All patients had a positive response to dopaminergic drugs. Cognitive decline, depression as well as rapid eye movement sleep behavior disorder were excluded for all subjects using the Mini Mental State Examination (cut-off score of 28), the Beck Depression Inventory-II (cut-off score of 6), and rapid eye movement sleep behavior disorder screening questionnaire (cut-off score of 5). Moreover, none of the patients reported having suffered from any neurological or psychiatric disorders other than idiopathic PD and none were taking, or stated to have ever been treated with antipsychotics or antidepressants drugs. The study was approved by the Local Ethics Committee (Comitato Etico Milano Area B). MRI data were obtained prospectively with written informed consent from all subjects. SPECT and clinical data were obtained retrospectively from patients' records.

### Spect with FP-CIT acquisition and analysis

Brain SPECT was performed on all patients 3 h after intravenous administration of 110–140 MBq of FP-CIT (DaTSCAN™, GE-Healthcare, UK), as described in Isaias et al. (2008). A historic group of 15 healthy volunteers, scanned on the same SPECT camera (4 males; median age: 67 years, range: 44–74 years), served as controls for SPECT measures (Isaias et al., 2007, 2008). Binding values of dopamine reuptake transporters (DAT) of the putamen and caudate nucleus were calculated on the basis of VOIs defined by means of the Basal Ganglia Matching Tool (Calvini et al., 2007). An asymmetry index was calculated for putamen, and analogously for the caudate nucleus as Isaias et al. (2008):

$$AI_{Putamen}\;=\;\left|\frac{\left(Putamen_{Ipsi}-\;Putamen_{Contra}\right)}{\left(Putamen_{Ipsi}+\;Putamen_{Contra}\right)}\;\times \;\;200\right|$$

where the subscript _*contra*_ (contralateral) refers to the side opposite to the clinically most impaired hemibody, and _*ipsi*_ (ipsilateral) refers to the most impaired hemibody. For HC we adopted the conventional of referring to the right side as ipsilateral.

### MR image acquisition

All subjects underwent MRI on a clinical 3T scanner (Achieva, Philips Medical Systems, Best, the Netherlands) with a 32-channel head coil. Conventional MRI scans were obtained to exclude coexisting central nervous system disorders. NM-MRI and multi-echo gradient echo scans were obtained for the comparisons of NM and iron with the SPECT data.

**Figure 1:**
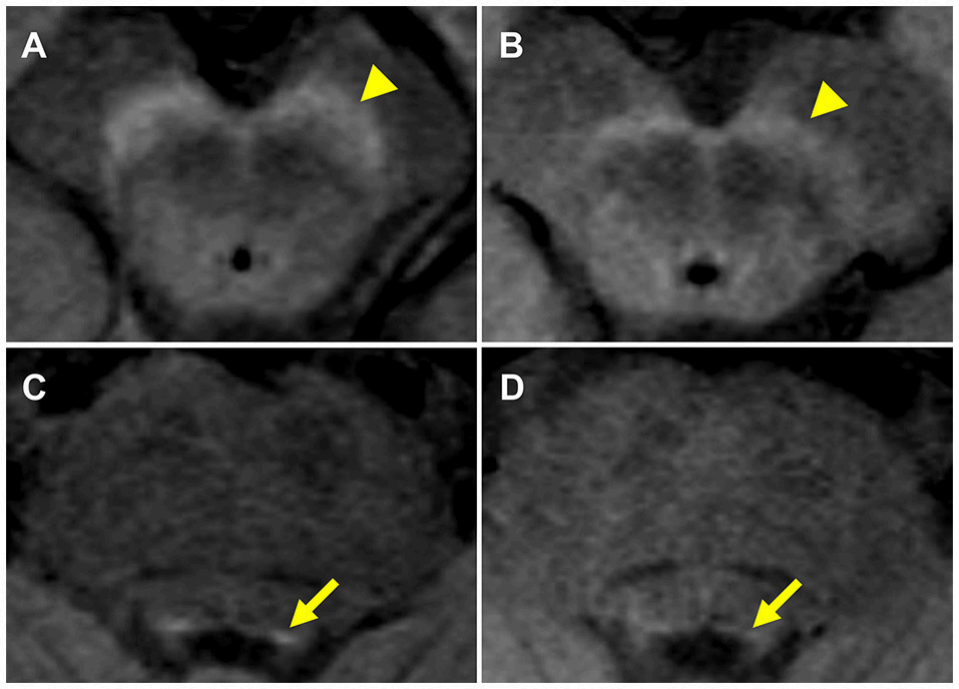
**NM-MRI of the substantia nigra and locus coeruleus**. NM-MRI images at the levels of the substantia nigra (arrowhead) and the locus coeruleus (arrow) of a 66-year-old healthy woman **(A,C)** and a 70-year-old man with PD **(B,D)**.

The NM-MRI scan consisted of a T1-weighted fast spin echo sequence with on-resonance magnetization transfer preparation pulses (TE/TR 12/670 ms, echo train length 4, field of view 216 × 164 mm^2^, acquisition/reconstruction resolution 0.5 × 0.6 × 3.0 mm^3^/0.5 × 0.5 × 3.0 mm^3^, 12 slices, five averages), with an acquisition time of approximately 7:40 min (Figure 1). As in previous studies (Nakane et al., 2008; Keren et al., 2009; Kitao et al., 2013; Ogisu et al., 2013; Chen et al., 2014), magnetization transfer preparation was used to improve the observable NM contrast. The oblique axial slices of the NM-MRI scan were defined perpendicular to the floor of the fourth ventricle, and covered approximately from the posterior commissure to the inferior pontine border.

Iron-sensitive imaging made use of a dual-echo, gradient echo sequence (TE1/TE2/TR 9.2/23/30.44 ms, field of view 240 × 240 mm^2^, resolution 1 × 1 × 1 mm^3^, 120 slices, flip angle 12°) with an acquisition time approximately 10 min. Magnitude and phase images were reconstructed from the gradient echo data for each echo time. Again, the sections were in the oblique axial plane perpendicular to the floor of the fourth ventricle.

### Neuromelanin-sensitive image analysis

**Figure 2:**
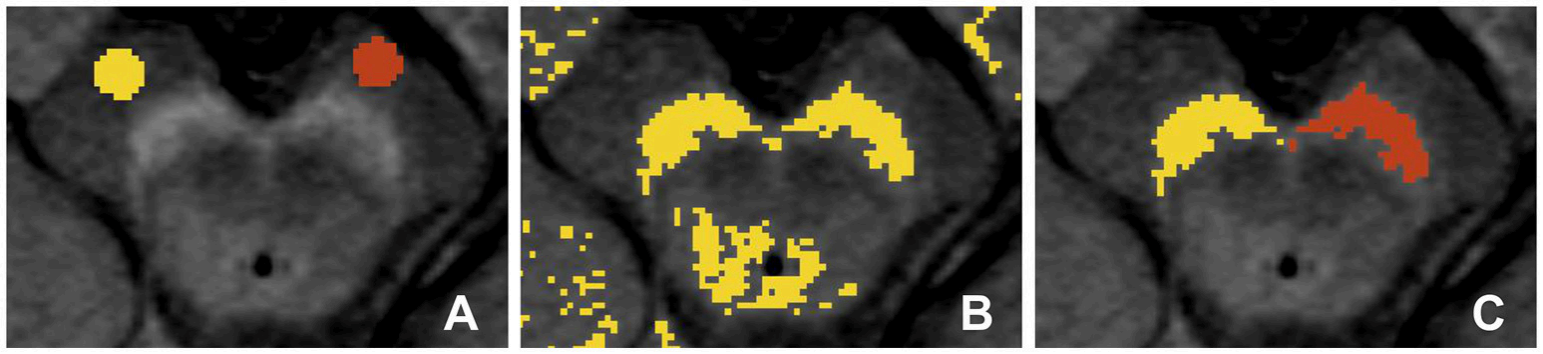
**Definition of region of interest (ROI) for the substantia nigra (SN) in the neuromelanin-MRI images involved (A) placing circular reference ROIs in the cerebral crus, (B) creating binary map to identify high intensity voxels and (C) isolation of the voxel clusters corresponding to the SN**.

As a measure of the presence of NM, the SN volume was estimated from the NM-MRI images using the 3D Slicer software package (version 4.3.1, http://www.slicer.org). In brief, similar to previous studies (Chen et al., 2014; Langley et al., 2015), a reader, blinded to the clinical status of the subjects, first defined circular (4 mm diameter) background regions of interest (ROIs) in the cerebral crus on the left and right sides (Figure 2A). This was repeated for four consecutive slices, in which the SN was visible. For each slice, a binary map was defined as the voxels in the mesencephalic region with signal intensity greater than:

$$\begin{array}{l} MN_{CC}\;+\;3\times SD_{CC} \end{array}$$

where *MN*_*CC*_ and *SD*_*CC*_ are the mean and standard deviation for the background ROI located in the cerebral crus on the corresponding slice and side (Figure 2B). ROIs for the SN were then defined on the binary map (Figure 2C).

The SN volume was calculated as the total number of voxels in the segmented SN multiplied by the voxel dimensions. The contrast-to-noise-ratio (CNR) between the SN and the cerebral crus was calculated for each slice and side of the SN as:

$$\begin{array}{l} CNR_{SN}\;=\frac{\left(MN_{SN}\;-\;MN_{CC}\;\right)}{SD_{CC}} \end{array}$$

where *MN*_*SN*_ and *MN*_*CC*_ correspond to the mean signal intensity of the SN and cerebral crus, respectively, and *SD*_*CC*_ corresponds to the standard deviation of the cerebral crus. The average CNR of the SN across slices was then calculated for each side. The asymmetry indices (AI_*SN-Volume*_ and AI_*SN-CNR*_) were calculated for the SN volume and the *CNR*_*SN*_ in a way analogous to that for SPECT measurements. Because the results of the above procedure may depend on the placement of the cerebral crus ROIs, the process of ROI drawing and calculation of the volume, CNR and AI values for the SN was repeated four times, and the average and standard deviation across measurements was calculated.

**Figure 3:**
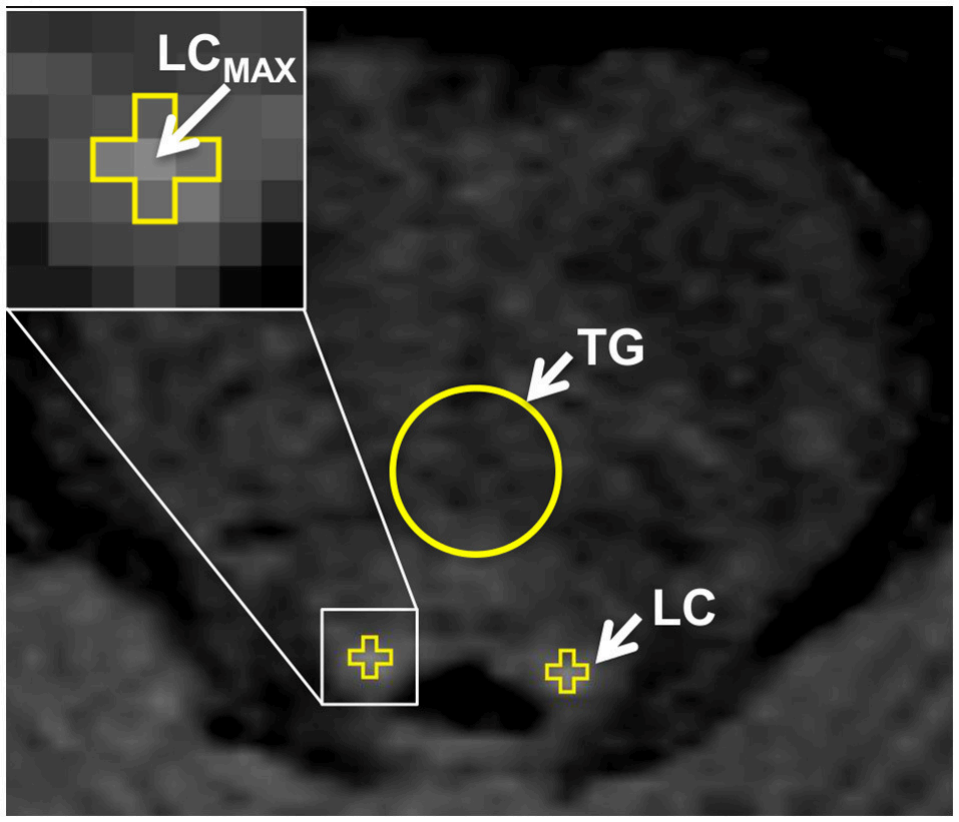
**Definition of the region of interest (ROI) for the locus coeruleus (LC) in the neuromelanin-MRI images involved identifying the highest intensity voxel adjacent to the fourth ventricle (LC_**MAX**_), which together with its four adjacent voxels were then used to calculate LC signal intensity**. The circular reference ROI in the pontine tegmentum is also indicated.

As the cross-sectional area of the LC is near, or possibly below, the resolution limit of our scans, we did not consider volumetry of LC to be reliable. We instead limited our evaluation of the LC to its contrast relative to surrounding tissue. For each side, the location of the LC was taken to be the highest intensity voxel adjacent to the fourth ventricle on that side (LC_MAX_) (Keren et al., 2009). Once each LC_MAX_ was located, the LC signal intensity was taken to be the intensity of LC_MAX_ and its four abutting voxels in the image plane (Figure 3). This was repeated for each of three consecutive 3 mm-thick axial slices. Background reference ROIs (circles with diameter 6 mm) were placed in the pontine tegmentum in the three slices in which the LC was identified. This processing was carried out using a custom software routine in Matlab (Mathworks Inc., Sherborn, MA, USA). The CNR between the LC and the pontine tegmentum (TG) was calculated in a way analogous to that for SN.

$$\begin{array}{l} CNR_{LC}\;=\frac{\left(MN_{LC}\;-\;MN_{TG}\;\right)}{SD_{TG}} \end{array}$$

Lastly, to allow direct comparisons between *CNR*_*SN*_ and *CNR*_*LC*_, measurements were normalized to the corresponding median value of the HC group to represent a % difference from normal value (%*CNR*_*SN*_ and %*CNR*_*LC*_, respectively).

### Iron-sensitive image analysis

Quantitative T2^*^ maps were calculated from the magnitude images at different echo times using the scanner manufacturer-supplied software, and inverted to obtain R2^*^ values. Phase and magnitude images for the different echo times were processed offline to calculate quantitative maps of magnetic susceptibility via the morphology-enabled dipole inversion method (MEDI-Toolbox for Matlab, Cornell MRI Research Lab, New York, NY, USA) (de Rochefort et al., 2010; Liu et al., 2011).

**Figure 4:**
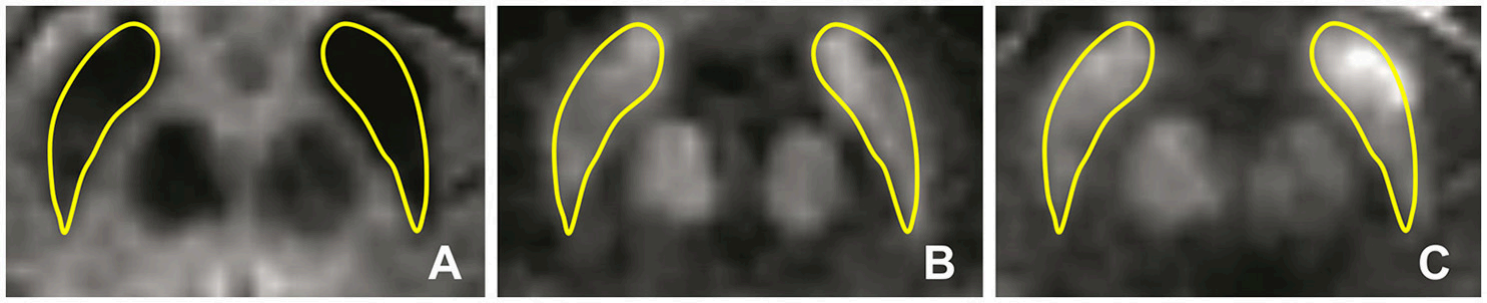
**Iron-sensitive imaging of the substantia nigra**. The regions of interest for the substantia nigra were defined bilaterally for iron-sensitive imaging as the hypointense band between the red nucleus and cerebral peduncle in the T2^*^-weighted images **(A)** and were then used to sample the R2^*^ maps **(B)** and susceptibility **(C)** maps, seen here for a 51-year-old man with PD.

Bilateral SN ROIs were delineated manually by a blinder researcher using the 3D Slicer software package. The SN ROIs were drawn on the gradient echo magnitude images with the longest echo-time (T2^*^ weighted), and then applied to the R2^*^ and susceptibility maps. The left and right SN were defined as the hypointense bands between the red nucleus and cerebral peduncle across six slices (Figure 4A). If the SN was visible in more than six slices, then the six central slices where the SN had the largest area were selected, starting either at the level of the red nucleus showing the largest radius, or one slice lower to minimize the probability of including the subthalamic nucleus. Subsequently, the ROIs were used to sample the R2^*^ (Figure 4B) and susceptibility (Figure 4C) maps, and the mean values of R2^*^ and susceptibility of the SN were calculated. The procedure was repeated two times, and the average of the two measurements was calculated.

### Statistical analysis

Statistical analysis were performed with the JMP statistical package, (version 10.0, SAS Institute, Inc., Cary, NC, USA). Gender difference between PD and HC groups was analyzed using Pearson's chi-squared test. Other differences between the PD and HC groups were analyzed by means of Mann-Whitney U test. The Spearman correlation coefficients (ρ) were calculated to investigate statistical dependencies amongst MRI and SPECT measures, demographic and clinical variables. The threshold level of statistical significance was set at *p* < 0.05 (False Discovery Rate corrected). A predictive analysis (least squares) between MRI and SPECT measures were then performed. Receiver operating characteristics analysis was performed to assess the diagnostic accuracy of NM-MRI results. The 95% confidence intervals for sensitivity and specificity were calculated according to the Clopper-Pearson method. The intraclass correlation coefficient was used to assess the intra-rater reliability.

## Results

### Clinical data

There were no significant gender or age differences between the PD patients and the HC group recruited for the present study. There was however, a significant difference in gender between the PD group and the pre-existing HC group (used here as controls for SPECT measures). Median age of the PD patients at motor symptoms onset was 56 years (38–70 years). Median disease duration was 6 years (3–15 years). Eight of the PD patients had disease durations greater than the median value of the PD group (11 years on average, range: 8–15 years); the remaining 10 patients had referred motor signs for 6 years or less (4 years on average; range: 3–6). All patients were at Hoehn and Yahr stage 2. The median UPDRS-III (motor part) score was 14 (range: 5–25) in a “meds-off” phase (12 h L-Dopa withdrawal of selegiline, rasagiline, amantadine, cabergoline, pergolide, and 72 h discontinuation of prolonged duration formulations of dopamine agonists). The median hemi-body UPDRS_*AK*_ score was 6 (range: 1–8) and 2 (range: 0–4) for the more and less affected side respectively. The average L-Dopa daily dose was 363 ± 152 mg and the average L-dopa equivalent daily dose was 502 ± 183 mg.

### SPECT with FP-CIT

When compared to a pre-existing group of HC, all PD patients had significantly reduced striatal DAT binding values in both putamen and caudate nucleus (Table 1). For both structures, the reduction was greater on the side contralateral to the most affected hemibody, as indicated by the asymmetry index (*AI*_*Putamen*_ and *AI*_*Caudate*_). As expected, DAT binding values of the Putamen_*contra*_ negatively correlated with disease duration (ρ = −0.51, *p* < 0.05), UPDRS-III and UPDRS_*AK*_ score (ρ = −0.46, *p* = 0.05 and ρ = −0.47, *p* < 0.05 respectively). It is worth noting that striatal DAT binding did not differ between male and female in our HC cohort.

**Table 1:** **Brain imaging findings in patients with PD and healthy subjects**.

		**Patients with PD**	**Healthy controls**	***p*-value**
**SPECT with FP-CIT**	**Putamen**_*contra*_	1.60 ± 0.67	4.60 ± 0.83	*P* < 0.001
	**Putamen**_*ipsi*_	2.06 ± 0.75	4.65 ± 0.83	*P* < 0.001
	**AI**_*Putamen*_	28.09 ± 22.08	2.39 ± 1.75	*P* < 0.001
	**Caudate**_*contra*_	2.97 ± 1.02	4.92 ± 0.98	*P* < 0.001
	**Caudate**_*ipsi*_	3.28 ± 0.97	4.94 ± 0.96	*P* < 0.001
	**AI**_*Caudate*_	20.84 ± 26.71	3.22 ± 2.33	*P* < 0.001
**NM-MRI**	**SN volume**_*-contra*_	236 ± 50 mm^3^	344 ± 51 mm^3^	*P* < 0.001
	**SN volume**_*-ipsi*_	283 ± 84 mm^3^	362 ± 60 mm^3^	*P* < 0.01
	**AI**_*SN-volume*_	21.88 ± 15.32	13.82 ± 8.22	*P* > 0.05
	**CNR**_*SN-contra*_	4.28 ± 0.41	4.91 ± 0.38	*P* < 0.001
	**CNR**_*SN-ipsi*_	4.42 ± 0.45	4.83 ± 0.37	*P* < 0.01
	**AI**_*SN-CNR*_	8.59 ± 7.25	6.08 ± 3.72	*P* > 0.05
	**CNR**_*LC-contra*_	3.54 ± 0.87	4.89 ± 0.61	*P* < 0.0001
	**CNR**_*LC-ipsi*_	3.85 ± 0.96	5.07 ± 0.47	*P* < 0.001
**Iron-sensitive MRI**	R2^*^_contra_	41.17 ± 10.96 s−1	37.47 ± 5.16 s−1	*P* > 0.05
	R2^*^_ipsi_	43.30 ± 10.01 s−1	39.09 ± 5.74 s−1	*P* > 0.05
	**Susceptibility**_*contra*_	0.16 ± 0.05 ppm	0.13 ± 0.03 ppm	*P* > 0.05
	**Susceptibility**_*ipsi*_	0.15 ± 0.05 ppm	0.13 ± 0.03 ppm	*P* > 0.05

*Data listed as mean ± standard deviation. PT, putamen; CN, caudate nucleus; AI, asymmetry index; Vol, volume; SN, substantia nigra; CNR, contrast-to-noise-ratio; contra, contralateral (to the side opposite to the clinically most impaired hemibody) and ipsi, ipsilateral. For HC we adopted the conventional of referring to the right side as ipsilateral*.

### Neuromelanin-sensitive MRI

Areas of hyperintensity were reliably detected in the NM-MRI images in positions corresponding to the locations of the SN and LC (Figure 1), consistent with previous NM-MRI and post-mortem studies (Sasaki et al., 2006; Keren et al., 2009, 2015; Kitao et al., 2013). To account for intra-rater variability, every measurement was performed four times, and the measurements were averaged. The intraclass correlation coefficients were 0.88, 074, and 0.74 for the SN volume, the *CNR*_*SN*_, and the *CNR*_*LC*_, respectively, which is an acceptable level of reproducibility. For all NM-MRI measures, there were no significant differences between sides (contra and ipsi) of the SN. In patients, the mean SN volume_*-contra*_ was lower than SN volume_*-ipsi*_, but the difference did not reach statistical significance (*p* = 0.08). In comparison to the HC group, the NM measures derived from NM-MRI were significantly reduced in PD patients (Table 1). SN volume_*-contra*_ (but not SN volume_*-ipsi*_) was negatively correlated with UPDRS-III (ρ = −0.61, *p* < 0.01) and UPDRS_*AK*_ (ρ = −0.63, *p* < 0.01).

**Figure 5:**
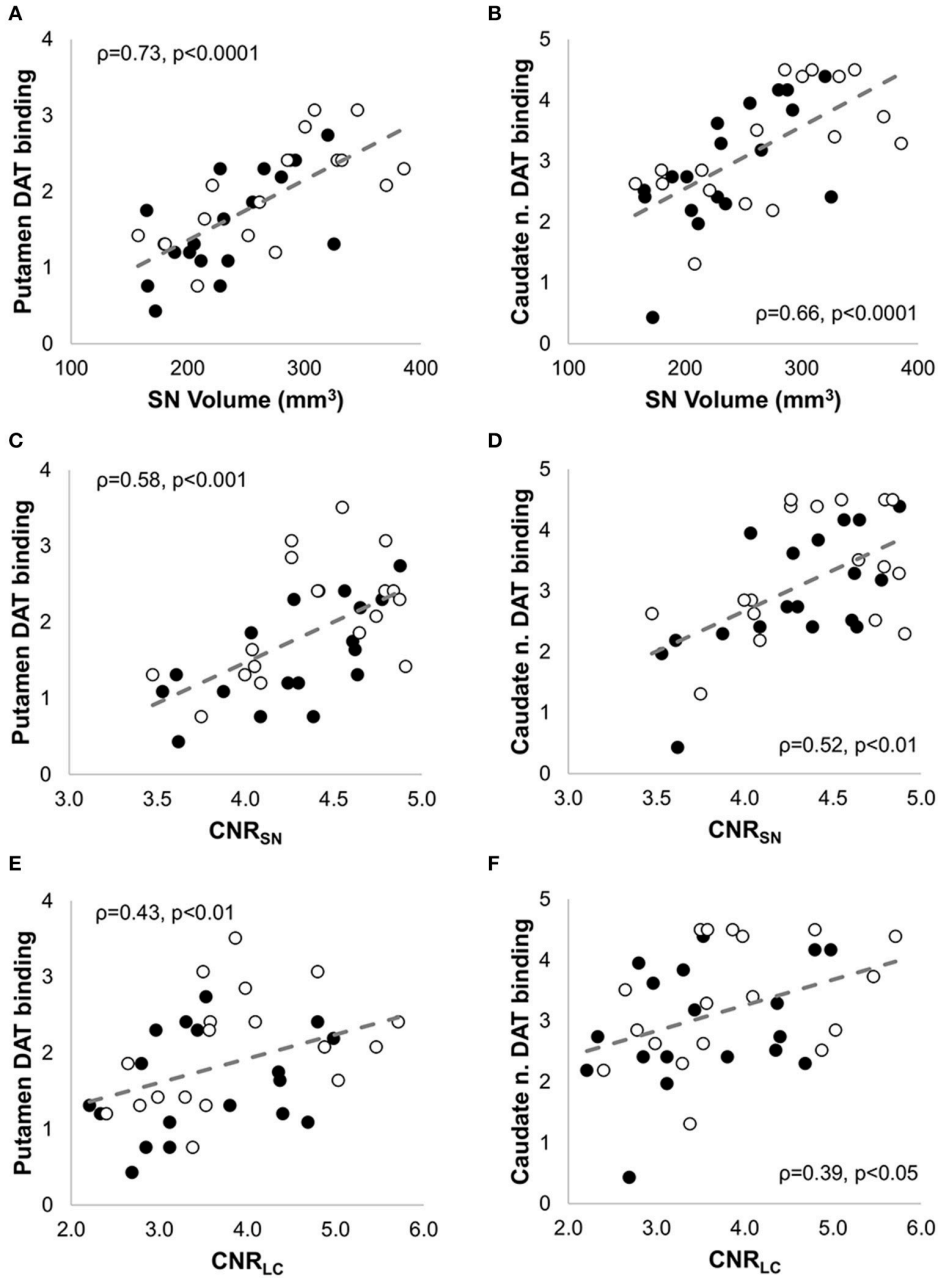
**Non parametric correlation analysis with linear regression between NM-MRI and SPECT measures in patients with PD**. NM-MRI measures of the substantia nigra (SN volume and *CNR*_*SN*_) and locus coeruleus (*CNR*_*LC*_) were significantly correlated with DAT binding values of the putamen **(A,C,E)** and caudate nucleus **(B,D,F)**. The Spearman correlation coefficients (ρ) and the *p*-values are indicated in each figure. The filled circles correspond to the measurements in the contralateral side, and the empty circles to the measurements in the ipsilateral side.

**Figure 6:**
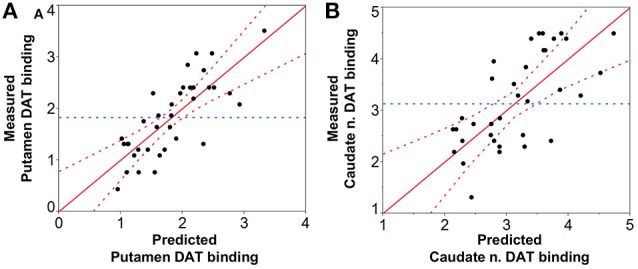
**Prediction analysis of DAT binding values of the (A) putamen and (B) caudate nucleus based on NM-MRI findings**. Prediction of DAT binding values was significant for both the putamen (*p* < 0.0001, *R*2 = 0.61, RMSE = 0.48, PRESS = 9.33) with SN volume being the best predictor (*p* < 0.0001) (*CNR*_*SN*_ leverage: *p* = 0.22, and *CNR*_*LC*_ leverage: *p* = 0.29), and the caudate nucleus (*p* < 0.0001, *R*2 = 0.50, RMSE = 0.73, PRESS = 21.53) again with SN volume as the best predictor (*p* < 0.01) (*CNR*_*SN*_ leverage: *p* = 0.11, and *CNR*_*LC*_ leverage *p* = 0.32). RMSE = Root mean square error; PRESS = Predicted residual error sum of squares.

*CNR*_*SN-contra*_ was positively correlated with *CNR*_*LC-contra*_ (ρ = 0.56, *p* < 0.05), and all but four PD patients showed greater %*CNR*_*LC-contra*_ values than %*CNR*_*SN-contra*_. AI_*SN-vol*_ and AI_*SN-*CNR**_ were not significantly different between PD and HC groups, and, for PD patients, they did not correlate with the corresponding AI_*Putamen*_ or AI_*Caudate*_ measurements. In fact, in seven PD patients the *CNR*_*SN*_ values were lower ipsilateral to the worst affected hemibody and opposite to the putamen with lowest DAT binding values. Despite this, significant positive correlation was also found between both SN volume_(*-contraand-ipsi*)_ (Figures 5A,B) and *CNR*_*SN*(*-contraand-ipsi*)_ (Figures 5C,D) and the corresponding DAT binding measurements of both the putamen and the caudate nucleus amongst the patients. The *CNR*_*LC*_ was also significantly correlated with the corresponding DAT binding measurements of putamen and caudate nucleus (Figures 5E,F). Of all measurements, the best predictor of DAT reduction of both the putamen and caudate nucleus was the SN volume (Figure 6).

The receiver operating characteristics analysis showed an area under the curve of 0.94, and provided three relevant cut-off values: (1) SN volume_*-contra*_ = 325 mm^3^ with 83% diagnostic accuracy, 100% sensitivity (95% confidence intervals: 81 to 100%), and 66.67% specificity (95% confidence intervals: 41 to 87%) in discriminating PD from HC (all PD subjects had lower values); (2) SN volume_*-contra*_ = 256 mm^3^ showing 83% diagnostic accuracy, 66.67% sensitivity (95% confidence intervals: 41 to 87%) and 100% specificity (95% confidence intervals: 81 to 100%) (all HC had higher values); (3) SN volume_*-contra*_ = 292 mm^3^, with the highest diagnostic accuracy and a good trade-off between sensitivity and specificity (accuracy = 86%, sensitivity = 89% (95% confidence intervals: 65.29 to 98.62%), and specificity = 83% (95% confidence intervals: 58.58 to 96.42%)).

### Iron-sensitive MRI

**Figure 7:**
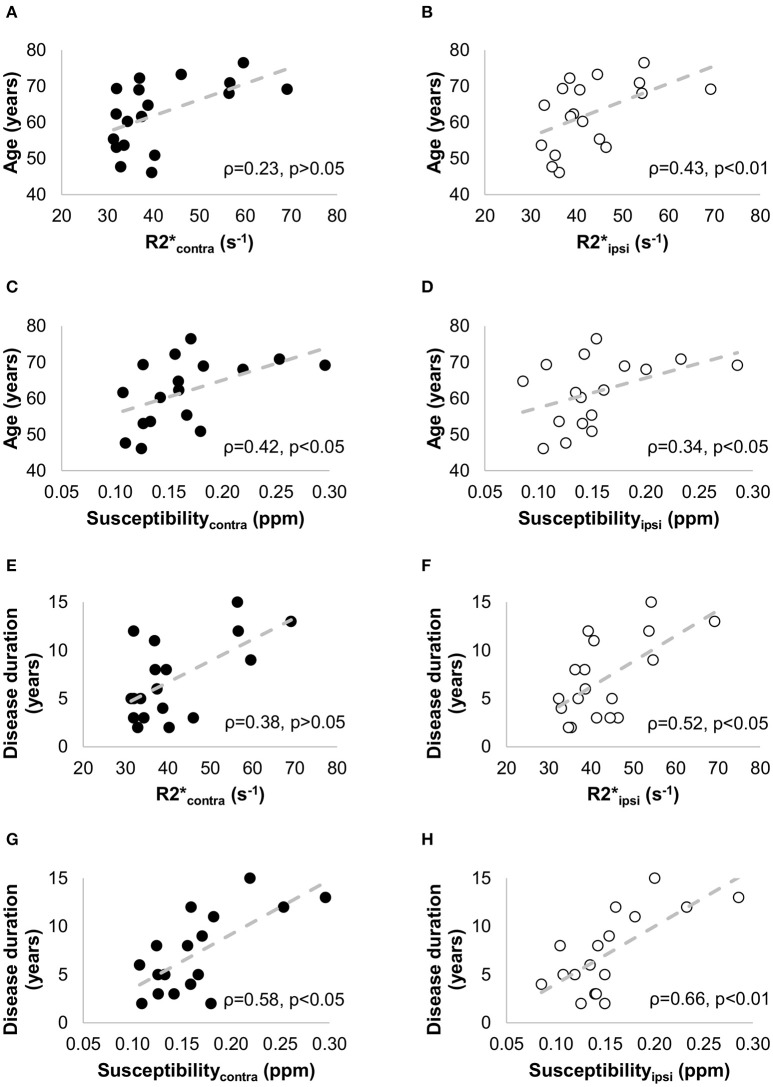
Non parametric correlation analysis with linear regression between the clinical data [age (A–D) and disease duration (E–H)] and the iron-sensitive imaging measures [R2^*^ (A,B,E,F) and susceptibility (C,D,G,H)] in -patients with PD. The Spearman correlation coefficients (ρ) and the *p*-values are indicated in each figure. The filled circles correspond to the measurements in the contralateral side, and the empty circles to the measurements in the ipsilateral side.

**Figure 8:**
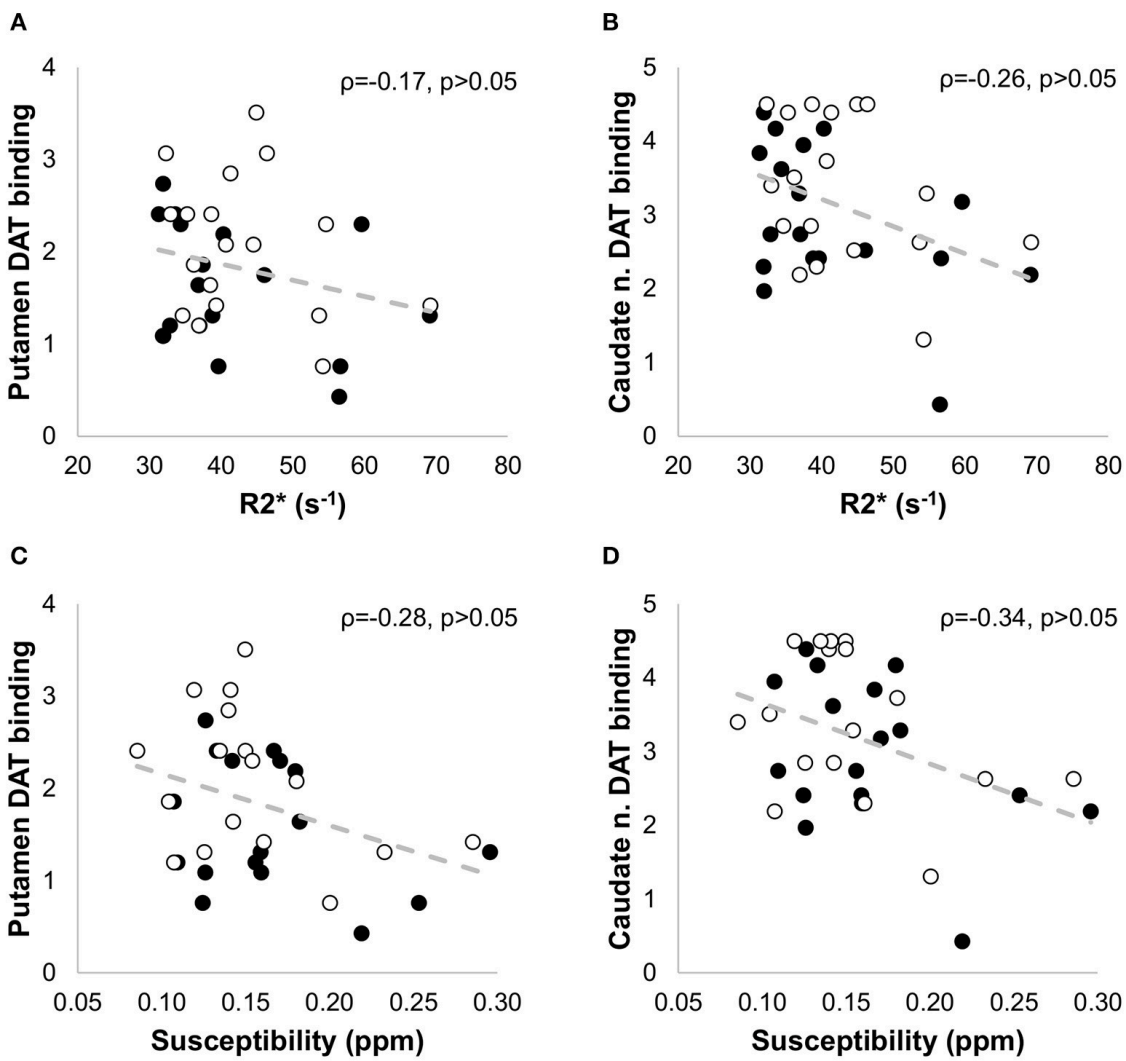
Non parametric correlation analysis with linear regression between the SPECT measures in the putamen (A,C) and caudate nucleus (B,D) and the iron-sensitive imaging measures (R2^*^ and susceptibility) in patients with PD. No significant correlation was found when comparing R2^*^ and susceptibility values with the SPECT findings. The Spearman correlation coefficients (ρ) and the *p*-values are indicated in each figure. The filled circles correspond to the measurements in the contralateral side, and the empty circles to the measurements in the ipsilateral side.

Quantitative maps of R2^*^ and susceptibility showed a large contrast between the SN and the surrounding brain tissue (Figure 4), but we did not find significant differences in R2^*^ and susceptibility values of the SN between the PD and HC groups (Table 1). In PD patients, positive correlations with age were seen for $\text{R}2_{ipsi}^{*}$ (ρ = 0.43, *p* < 0.01), Susceptibility_*ipsi*_ (ρ = 0.34, *p* < 0.05), and Susceptibility_*contra*_ (ρ = 0.42, *p* < 0.05), and with disease duration for $\text{R}2_{ipsi}^{*}$ (ρ = 0.52, *p* < 0.05), Susceptibility_*ipsi*_ (ρ = 0.66, *p* < 0.01) and Susceptibility_*contra*_ (ρ = 0.58, *p* < 0.05) (Figure 7). Neither R2^*^ nor susceptibility values correlated with any other demographic or clinical parameter. Of the two correlated variables (i.e., age and disease duration), disease duration proved, at a Stepwise regression analysis, to independently correlate with both parameters ($\text{R}2_{ipsi}^{*}$: F-ratio = 4.22, *p* = 0.05; Susceptibility_*ipsi*_: F-ratio = 15.48, *p* < 0.01), whereas the correlation with age was not statistically significant. Lastly, no significant correlation was found when comparing R_2_* and susceptibility values with NM-MRI or SPECT findings (Figure 8). The intraclass correlation coefficient for the measurements were 0.91 and 0.90 for the R2^*^ and susceptibility, respectively.

## Discussion

PET and SPECT have been extensively employed to elucidate the functional changes associated with PD and other neurodegenerative disorders (Isaias et al., 2007, 2011; Brooks and Pavese, 2011; Stoessl et al., 2011, 2014). To date, the imaging measures of dopaminergic function by SPECT with FP-CIT represent a robust biomarkers for early PD detection and assessing disease progression. Although the deployment of PET and SPECT scanners is becoming more common, their availability is still limited. In addition, the expense, duration, and invasive nature of radionuclide imaging limit its clinical application, particularly for longitudinal assessment, large cohorts of patients, and evaluation in non-confirmed cases or subjects at risk to develop PD. MRI has been gaining ground over the past decade as an attractive alternative to radiotracer methods. MRI techniques have evolved to provide novel information about the degenerative process in PD and other movement disorders opening potential applications for the differential diagnosis. Its non-invasive nature, diversity of possible contrasts, lower cost, good availability, and in particular the fact that MRI does not use ionizing radiation contribute to its attractiveness.

Recently, the NM-MRI (Sasaki et al., 2006), has provided notable contrast between NM-containing structures and surrounding brain tissues with potential applications as biomarker of PD. Several NM-MRI studies (Shibata et al., 2006; Schwarz et al., 2011; García-Lorenzo et al., 2013; Miyoshi et al., 2013; Ohtsuka et al., 2014; Castellanos et al., 2015) have shown significant reductions in measures of NM-MRI contrast and volume of the SN and LC in PD patients compared with healthy controls, leading to the suggestion that the NM-related contrast in MRI images reflects a loss of NM-containing neurons. In fact, a direct comparison between post-mortem NM-MRI and neuropathological findings (Kitao et al., 2013) has shown that NM-MRI signal intensity in the SNc is closely associated with the quantity of NM-containing neurons, supporting the link between NM-MRI hyperintensity with the presence of NM, and raising the prospect of NM-MRI being a potential biomarker of PD.

In this study we investigated the correlations between MRI markers of the SN and LC of PD patients and the corresponding nigro-striatal dopaminergic innervation loss as measured by SPECT with FP-CIT. Both of the examined NM-MRI based measures of the SN (SN volume and *CNR*_*SN*_) correlated significantly with dopaminergic striatal innervation loss as measured by SPECT with FP-CIT (Figure 5) and SN volume was highly correlated with striatal DAT binding values (Figure 6). Also of relevance, NM-MRI measurements, as well as SPECT findings, correlated with the severity of PD-related motor signs. Our results support the ability of NM-MRI to differentiate PD patients from healthy subjects as indicated in previous reports (Sasaki et al., 2006; Ohtsuka et al., 2014). Also in agreement with prior NM-MRI studies, PD patients showed significant reductions in SN contrast and volume. In particular, the cut-off of SN volume_*-contra*_ = 292 mm^3^ obtained by receiver operating characteristics analysis provided a diagnostic accuracy of 86%, with good sensitivity (89%) and specificity (83%).

The discrepancy between asymmetry indexes (i.e., AI_*Putamen*_ and AI_*SN-volume*_ or AI_*SN-CNR*_) was unexpected. In particular, the asymmetry of DAT binding measurement at a striatal level in PD patients was not mirrored by an asymmetry of NM-MRI measurements. In light of the close correlation between post-mortem NM-MRI and the quantity of NM-containing neurons (Kitao et al., 2013), our findings for AI_*SN-volume*_ and AI_*SN-CNR*_ may reflect the actual SN neuron loss, whereas the asymmetric striatal DAT density seen with AI_*Putamen*_, and the consequent asymmetry of clinical signs in PD patients, might be influenced by pre-synaptic compensatory mechanisms.

Under the assumption that %*CNR*_*LC*_ reflects neuron loss in the LC area, the finding of greater %*CNR*_*LC*_ in comparison to the %*CNR*_*SN*_, would support the presence of an ascending pathological process in PD patients (Braak et al., 2003). This was indeed evident in 14 patients. However, the fact that four patients did not show such a pattern suggests that it does not occur in all PD patients. Notably, a reduced %*CNR*_*LC*_ (averaged for both hemispheres) from NM-MRI has recently been observed to be specific to PD patients with concomitant rapid eye movement sleep behavior disorder (García-Lorenzo et al., 2013). Thus, the clinical characteristics and evolution of specific risk factors may be determined by the changes at the LC, but require studies suitable for the identification of such relationships. Despite these interesting results, the difficulty in distinguishing the LC from the sub-coeruleus region due to its position and size, and the technical limitations of our NM-MRI (see below), we consider the imaging and analysis methods for this structure to be unsatisfactory.

The process of iron accumulation in SN of PD patients is also not completely understood. Iron concentrations in dissected SN pars compacta and pars reticulata measured with accurate spectroscopic methods have shown that iron concentrations in these regions increase with disease severity (Dexter et al., 1987; Hirsch et al., 1991), and it has been proposed that high iron content in the SN makes this region susceptible to neurodegeneration (Zecca et al., 2004; Ward et al., 2014). In the literature however, iron imaging results are mixed. Several reports have described an increase iron concentration in the SN of PD patients (Graham et al., 2000; Martin et al., 2008; Baudrexel et al., 2010), but equally, we and others did not find significant differences in iron concentration between PD and controls (Galazka-Friedman et al., 1996; Zecca et al., 2004; Ward et al., 2014). We did however, find greater iron accumulation, as indicated by R2^*^ and susceptibility values, in PD patients with longer disease duration, even accounting for age, and regardless of their disease severity or dopaminergic drug doses. Moreover, across the available MRI studies a rather large range of iron concentration and confidence intervals has been reported both in PD patients and controls (Martin et al., 2008; Lotfipour et al., 2012; Ulla et al., 2013). These observations lead us to suspect that subtle differences in patient characteristics and measurement technique may be responsible for the inconsistency of results obtained with iron imaging by MRI, such that better standardization of technique is needed if the limitations of MRI for accurate measurement of iron in brain tissue are to be overcome and it is to have a role as a biomarker for PD (Martin et al., 2008).

We did not attempt nigral subdivision into pars compacta and pars reticulata, such as can be performed at higher magnetic field (Lotfipour et al., 2012; Lehéricy et al., 2014). The region of interest definitions we have used are however strongly tied to the contrast in the images. Differences in location and morphology of the SN are apparent in the ROIs defined for our NM- and iron-sensitive MRI. Whereas areas of hyperintensity were detected in the NM-MRI, the T2^*^ weighted images showed the SN as a hypointense band, rostral, and lateral to the hyperintense area in the NM images. Langley et al. have recently found NM and susceptibility weighted imaging contrasts to be selectively sensitive to caudal and rostral compartments of the SN respectively (Langley et al., 2015). They proposed that the two histologically subregions of the SN, the SN pars compacta and the SN pars reticulata, are delineated by NM-MRI and susceptibility weighted imaging, respectively. NM-MRI and susceptibility weighted imaging (or QSM as used herein) are also complementary in their respective sensitivities to neuronal death and iron deposition, such that investigation of the degenerative processes in the SN is likely to benefit from their combined use.

This study presented several limitations. First, the number of patients in our study did not allow us to cover the full spectrum of PD stages, and we selectively recruited patients without any (non-dopaminergic) PD-related comorbidities (e.g., dementia, depression, postural instability or falls, rapid eye movement sleep behavior disorder, etc.). This may limit our scope for establishing clinical correlations, but was suited to the study design, which was to directly compare MRI metrics of SN pathology and its consequent striatal dopaminergic innervation loss. Second, we obtained cut-off values for SN volume showing high sensitivity and specificity in identifying PD patients, but further studies testing these values in other groups of PD patients and HC are required to validate their usability to differentiate PD patients from HC. The estimates of SN volume, and thus the cut-off points are dependent on the segmentation method and imaging sequence characteristics. In this study, we used a semiautomatic segmentation method based on the approach previously described (Schwarz et al., 2011; Chen et al., 2014). Although our intra-operator variability in segmentation was reasonable, this and the inter-operator variability due to background ROI positioning could be largely eliminated in future studies through the use of completely automated segmentation for quantification of SN and LC volumes using NM-MRI, as recently suggested (Castellanos et al., 2015). Third, the NM-MRI method applied here involved a clinically acceptable scan time below 10 min, but did not optimally resolve the LC for volumetric assessment and even the SN volume measurements were subject to partial volume effects. Further, the NM-MRI 2D multislice scan yielded inhomogeneous signal intensities between slices due to its sensitivity to radiofrequency field inhomogeneities, and differing magnetization transfer weighting through cross-talk effects. These necessitated a slice by slice approach to ROI definition. A 3D acquisition, as described by Ogisu et al. (2013), may allow more robust ROI definition. Last, there were also some limitations regarding the iron-sensitive MRI technique. A greater number of echoes may improve the R2^*^ estimation and the quality of the QSM results.

In conclusion, NM-MRI is a means of quantifying SN pathology in PD patients that closely correlates with dopaminergic striatal innervation loss. It may serve as an imaging marker of PD, in particular regarding SN neuron loss, although further longitudinal multi-imaging studies, possibly involving subjects at risk of PD, are required.

## Author contributions

II, PS, GP, LZ, AC designed research; II, PT, PS, GM, AC performed research; II, PT, PS, GM, LM, AC analyzed data; II, PT, PS, GP, LZ, AC wrote the manuscript.

## Funding

This study was sponsored by Fondazione IRCCS Ca' Granda Ospedale Maggiore Policlinico di Milano, the Grigioni Foundation for Parkinson's Disease, and the Interdisziplinäres Zentrum für Klinische Forschung (IZKF) of the University Hospital Wuerzburg.

### Conflict of interest statement

The authors declare that the research was conducted in the absence of any commercial or financial relationships that could be construed as a potential conflict of interest.
